# Draft genome sequence of *‘Treponema phagedenis’* strain V1, isolated from bovine digital dermatitis

**DOI:** 10.1186/s40793-015-0059-0

**Published:** 2015-09-21

**Authors:** Mamoona Mushtaq, Shahid Manzoor, Märit Pringle, Anna Rosander, Erik Bongcam-Rudloff

**Affiliations:** Department of Animal Breeding and Genetics Science, Swedish University of Agricultural Science, SLU-Global Bioinformatics Centre, Uppsala, SE 750 07 Sweden; Department of Animal Health and Antimicrobial Strategies, National Veterinary Institute, Uppsala, SE 751 89 Sweden; Department of Biomedical Sciences and Veterinary Public Health, Swedish University of Agricultural Sciences, Uppsala, SE 750 07 Sweden; Department of Information Technology, University of the Punjab, Lahore, Pakistan

**Keywords:** *‘T. phagedenis’*, Genome assembly, Digital dermatitis, Västra Götaland, Sweden

## Abstract

*‘Treponema phagedenis’* is considered to be a key agent in the pathogenesis of bovine digital dermatitis, an infectious foot condition of economic and animal welfare importance. We hereby report the draft sequence of *‘T. phagedenis’* strain V1. The draft genome assembly consists of 51 scaffolds comprising 3,129,551 bp and a GC-content of 39.9 %. Putative pathogenicity related factors have been identified in the genome that can be used in future studies to gain insight into the pathogenic mechanisms of ‘*T. phagedenis*’.

**Electronic supplementary material:**

The online version of this article (doi:10.1186/s40793-015-0059-0) contains supplementary material, which is available to authorized users.

## Introduction

Digital dermatitis is a painful infection of the foot and is the leading cause of lameness in dairy cattle. Secondary effects of lameness are decreased milk production and weight loss leading to economic losses and animal welfare problems [1]. The disease is characterized by a diffuse or circumscribed superficial dermatitis of the skin at the coronary margin of the hoof. Erosive lesions are formed at the superficial layer of epidermis accompanied by pain, swelling and foul odor. Bacteria from different genera have been identified from these lesions, among them spirochetes of the genus *Treponema* are most prevalent [2–4]. Members of this genus constitute both commensal and pathogenic spirochetes. *Treponema pallidum*, which causes syphilis, is a well-known example of a pathogenic treponeme. A *Treponema* phylotype recently suggested being the same species as is the human commensal *‘**Treponema phagedenis**’* [5] which is considered to be a key agent in the pathogenesis of digital dermatitis [6–9]. *‘**T. phagedenis**’* is thought to be important for lesion development because it is found at the interface with healthy tissue [10] and has been detected in infected cattle from Europe [11], North America [12], and Asia [13]. To identify the putative pathogenicity related factors of ‘*T. phagedenis*', we performed sequencing of the ‘*T. phagedenis*’ strain V1 chromosome [14].

## Organism information

### Classification and features

**Fig. 1:**
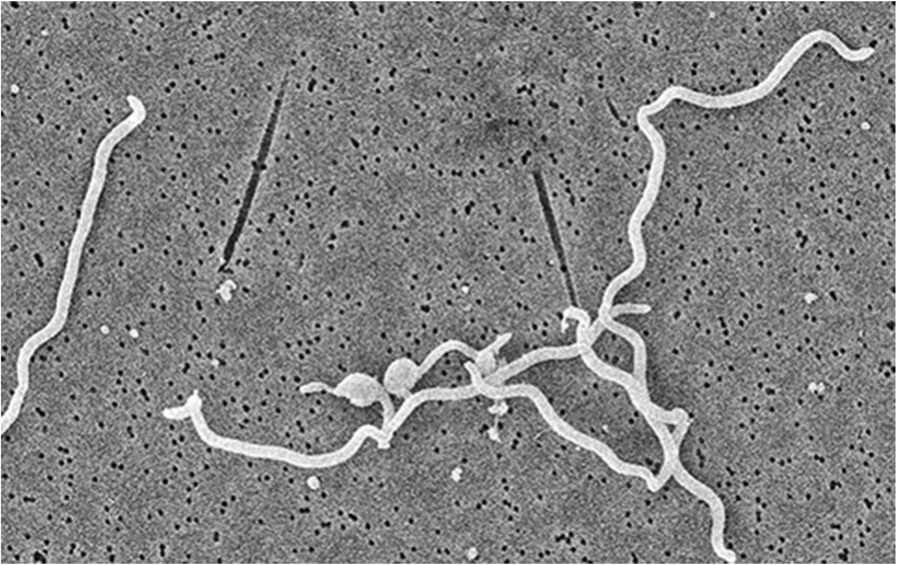
A scanning electron microscope picture of *Treponema phagedenis* V1 cells. Photo: Leif Ljung

**Fig. 2:**
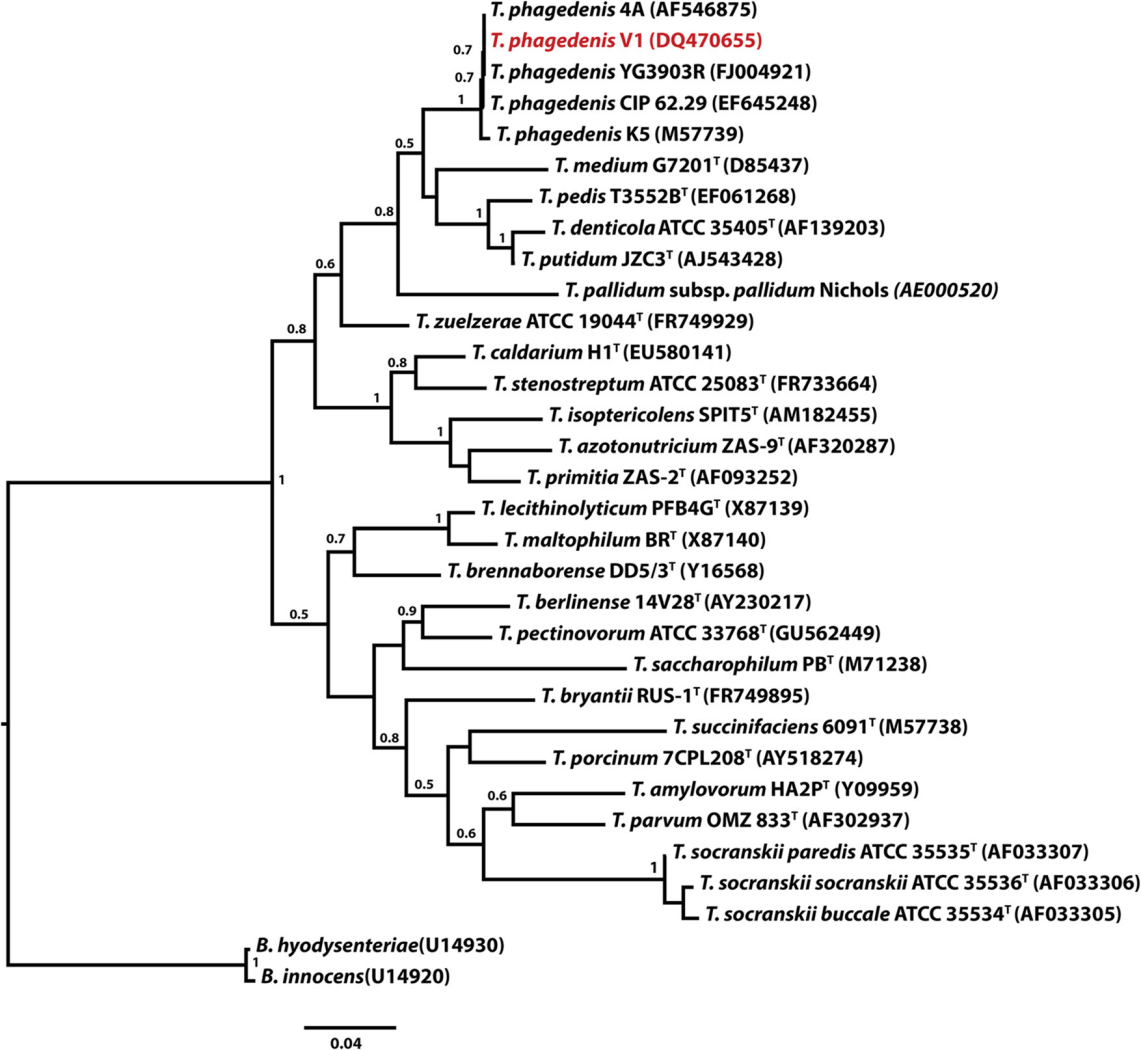
16S rRNA phylogenetic tree; Phylogenetic tree of 16S rRNA sequences highlighting the position of ‘*Treponema phagedenis*’ strain V1 relative to other ‘*Treponema phagedenis’* strains and to the other species within the genus. *Brachyspira hyodysenteriae* and *Brachyspira innocens* are used as out-group. The evolutionary history was inferred from 1212 aligned characters [42, 43]. The tree is drawn to scale, with branch lengths measured in the number of substitutions per site. Numbers above branches are support values from 1000 bootstrap replicates. 0.04 on the scale bar represents 4 substitutions in 100 bp. Evolutionary analyses were conducted using maximum Likelihood method in MEGA6 [44]

'*Treponema phagedenis**'* strain V1 (Fig. 1) was isolated from a Swedish dairy cow [14]. Strains 4A and YG3903R were isolated from digital dermatitis lesion in cattle from USA and Japan respectively [12, 13]. According to 16S rRNA sequence comparison using NCBI blast [15] *‘**T. phagedenis**’* V1 (DQ470655) shares 100 % identity with *‘**T. phagedenis**’* strains 4A (AF546875) and YG3903R (FJ004921) and 98 %-99 % identity with human strains CIP 62.29 (EF645248) and K5 (M57739). Among other treponemes, *‘**T. phagedenis**’* V1 is most closely related to *Treponema putidum* (AJ543428) and *Treponema denticola* (AF139203) sharing 93 % 16S rRNA identity with them. Figure 2 shows the phylogenetic relationship of *‘**T. phagedenis**’* V1 with the other *Treponema* species in a 16S rRNA based tree.

‘*Treponema phagedenis*’ is a helically, right-handed coiled bacterium with bent ends that are motile [16]. The typical size of ‘*T. phagedenis*’ ranges in length from 0.8 to 15 μm and 0.3 to 0.4 μm in width, with 7 to 9 flagella attached on each end [5, 12]. These bacteria are mostly host-associated, anaerobic and have fastidious growth requirements. *‘**Treponema phagedenis**’* strain V1 was isolated from a clinical sample from a digital dermatitis lesion. [14]. The sample was taken from an acute lesion in a herd with continuous problems with digital dermatitis. According to the API ZYM profile, *‘**T. phagedenis**’* strain V1 shows a positive reaction for alkaline phosphatase, C_4_ esterase, C_8_ esterase lipase, acid phosphatase, naptholphosphohydrolase, β-galactosidase, and N-acetyl-β-glucosaminidase. The antimicrobial susceptibility test performed on *‘**T. phagedenis**’* strain V1 shows that it is susceptible to tiamulin, valnemulin, tylosin, aivlosin and doxycycline [14]. Also, three immunogenic proteins, TmpA, Ttm, and PrrA, have been detected in *‘**T. phagedenis**’*. The presence of antibodies against these proteins has been identified in high titer in sera from cattle with digital dermatitis through indirect enzyme-linked immunosorbent assay [17]. General features of *T. phagedenis* V1 are stated in Table 1.

**Table 1:** Classification and general features of *‘Treponema phagdenis’* strain V1 [33]

MIGS ID	Property	Term	Evidence code^a^
	Classification	Domain *Bacteria*	TAS [34]
		Phylum *Spirochaetes*	TAS [35]
		Class ‘*Spirochaetia’*	TAS [36]
		Order *Spirochaetales*	TAS [37–39]
		Family *Spirochaetaceae*	TAS [40]
		Genus *Treponema*	TAS [6, 14]
		Species ‘*Treponema phagdenis’*	TAS [5, 14]
		Strain: *V1*	
	Gram stain	negative	TAS [41]
	Cell shape	Helical	TAS [41]
	Motility	Motile	TAS [14, 17]
	Sporulation	Non-sporulating	NAS
	Temperature range	30-42 °C	NAS [41]
	Optimum temperature	37 °C	NAS [41]
	pH range; Optimum	6–8.5; 7	TAS [5]
	Carbon source	D-glucose	IDA
MIGS-6	Habitat	Digital dermatitis lesion in cattle	TAS [14]
MIGS-6.3	Salinity	Not reported	
MIGS-22	Oxygen requirement	Anaerobic	NAS
MIGS-15	Biotic relationship	Host-associated	NAS
MIGS-14	Pathogenicity	Potential pathogen in cattle	TAS [14]
MIGS-4	Geographic location	Västra Götaland county, Sweden	TAS [14]
MIGS-5	Sample collection	2005	TAS [14]
MIGS-4.1	Latitude	Not reported	
MIGS-4.2	Longitude	Not reported	
MIGS-4.4	Altitude	Not reported	

*IDA* Inferred from Direct Assay, *TAS* Traceable Author Statement (i.e., a direct report exists in the literature), *NAS* Non-traceable Author Statement (i.e., not directly observed for the living, isolated sample, but based on a generally accepted property for the species, or anecdotal evidence). These evidence codes are from the Gene Ontology project [33]

^a^Evidence codes

## Genome sequencing information

### Genome project history

*‘**Treponema phagedenis**’* strain V1 was selected for sequencing in 2009 at the Swedish University of Agricultural Sciences (SLU), Uppsala, Sweden. The genome was assembled and annotated by the SLU-Global Bioinformatics Centre at SLU. The genome project is deposited in the Genomes OnLine Database [18] with GOLD id Gi0072982 and the draft genome assembly is deposited in the European Nucleotide Archive database with accession number (CDNC01000001-CDNC01000051) under the study accession number: PRJEB5300. The aim of the sequencing was to identify genes that are linked to pathogenicity and virulence in related bacteria, to strengthen the hypothesis that bacteria of the genus *Treponema* causes digital dermatitis in cattle. Almost nothing is known about virulence factors of treponemes involved in digital dermatitis. Table 2 contains the summary of the project information.

**Table 2:** Project information

MIGS ID	Property	Term
MIGS 31	Finishing quality	Draft
MIGS-28	Libraries used	454 Single end reads, Illumina paired end reads
MIGS 29	Sequencing platforms	454, Illumina hiseq
MIGS 31.2	Fold coverage	25×, 100×
MIGS 30	Assemblers	Newbler
MIGS 32	Gene calling method	Prodigal
	Locus Tag	TPHV1
	GeneBank ID	CDNC00000000
	GenBank Date of Release	18-01-2015
	GOLD ID	Gp0092386
	BIOPROJECT	PRJEB5300
MIGS 13	Source Material Identifier	Not reported
	Project relevance	Potential pathogen

### Growth conditions and genomic DNA preparation

*'**Treponema phagedenis**'* V1 was grown in flasks containing 10 ml FABGS (LAB071 fastidious anaerobe broth, LabM, with 2.0 g D-glucose per liter and 25 % fetal calf serum, S 0115, Biochrom AG), and incubated in anaerobic jars at 37 °C, 90 rpm. Genomic DNA was prepared with the DNeasy Blood & Tissue Kit (QIAGEN) following the protocol for Gram-negative bacteria [17]. The DNA concentration measured by Picodrop Microliter UV/Vis Spectrophotometer was 566 ng μl^−1^.

### Genome sequencing and assembly

The genomic sequence was obtained using a combination of Roche 454 GS FLX sequencing platform at the Royal Institute of Technlogy in Stockholm and Illumina HiSeq 2000 at the Uppsala sequencing platform. For Illumina sequencing three different libraries were used with the insert size of 160 bp, 305 bp and 505 bp. A total of 306,592 reads with the average read length of 300 bp were obtained from 454 sequencing and 60,174,091, 61,097,083, and 71,967,626 reads from the 160, 305 and 505 bp insert size libraries, respectively, from the Illumina sequencing. Subsets of reads from all three libraries were generated using a custom perl script to lower the coverage before performing assembly. Four different assemblies were produced, these include (i) hybrid assembly of 454 reads and Illumina reads from 160 bp insert size library (ii) hybrid assembly of 454 reads and Illumina reads from 305 bp insert size library (iii) hybrid assembly of 454 reads and Illumina reads from 505 bp insert size library (iv) 454 reads assembly. The resulting assemblies varied in size from 2.9 to 3.1 Mbp with the average GC content of 39 %. Assembly was performed with the GS de novo assembler version 2.5.3 (Roche) using reads from each Illumina paired end library and the 454 sequencing. Resulting assemblies were compared using the MAUVE genome alignment tool [19]. The hybrid assembly produced from 454 reads and Illumina reads from 305 bp insert size library was selected for further analysis. Selection was based on N50 statistics, number of contigs and the length of the largest contig. Assembly statistics of all assemblies are provided in supporting Additional file 1: Table S1. Scaffolding of the selected assembly was performed using SSPACE [20] and possible removal of gaps present in scaffolds was done using Gapfiller [21] and. Homopolymer errors were corrected manually using Consed [22].

### Genome annotation

The structural and functional annotation was accomplished via the Magnifying Genome (MaGe) Annotation Platform [23]. Prediction of tRNA and rRNA genes was performed using tRNAscan-SE version 1.23 [24] and RNAmmer version 1.2 [25], respectively. Putative functions of the encoding genes were assigned automatically by MAGE′s inbuilt BlastP searches against the UniProt and Trembl, TIGR-Fam, Pfam, PRIAM, COG and InterPro databases. Putative phage prediction was performed using PHAST (**PHA**ge **S**earch **T**ool) webserver [26]. Proteins with signal peptides were predicted using SignalP v 4.1 [27] and TMHMM Server, v.2.0 [28] was used to predict transmembrane helices in the protein sequences.

## Genome properties

**Fig. 3:**
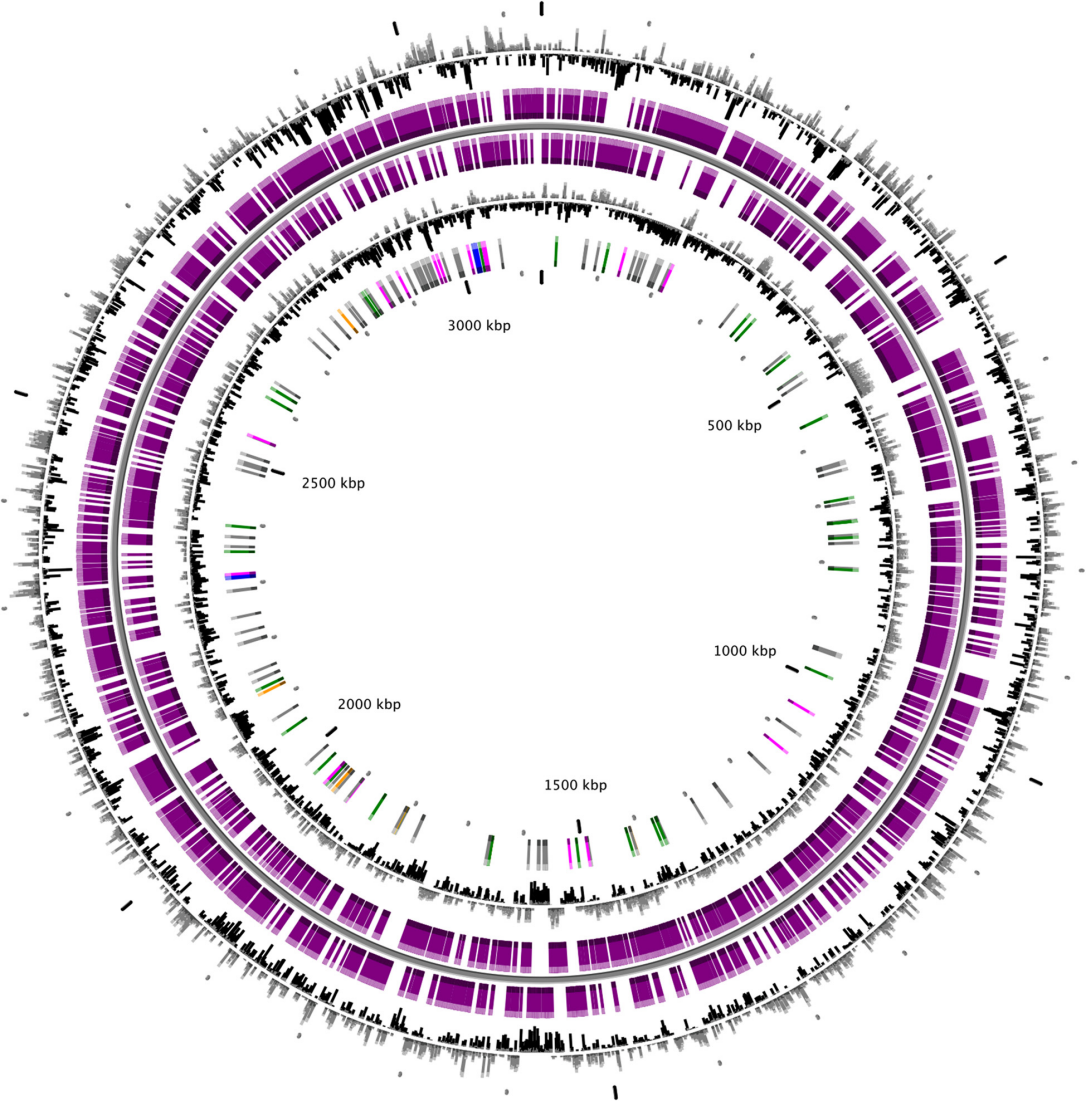
Circular representation of genome; Circular map (from the outside to the center): (1) GC percent deviation (GC window - mean GC) in a 1000-bp window. (2) Predicted CDSs transcribed in the clockwise direction. (3) Predicted CDSs transcribed in the counterclockwise direction. (4) GC skew (G + C/G-C) in a 1000-bp window. (5) rRNA (blue), tRNA (green), miscRNA (orange), Transposable elements (pink) and pseudogenes (grey)

The draft genome assembly comprised 60 contigs in 51 scaffolds with a total size of 3,129,551 bp (Fig. 3) that corresponds well to the size of two previously sequenced ‘*T. phagedenis**’* strains, 4A isolated from bovine digital dermatitis and F0421 isolated from human urogenitalia, with the assembly sizes of 3,027,773 and 2,830,421 respectively. The G + C content of the assembly was 39.9 %. In total 3,222 genes were predicted, of which 3,157 were protein coding genes. Table 3 contains the general genomic features. The classification of the protein coding genes in different COG categories is shown in Table 4.

**Table 3:** Genome statistics

Attribute	Value	% of Total
Genome size (bp)	3,129,551	100.0
DNA coding (bp)	2,623,392	83.8
DNA G + C (bp)	1,249,392	39.9
DNA scaffolds	51	100.0
Total genes	3,222	100.0
Protein coding genes	3,157	98
RNA genes	51	1.6
Pseudo genes	9	0.3
Genes in internal clusters		
Genes with function prediction	1,547	48
Genes assigned to COGs	2,051	63.7
Genes with Pfam domains	1,788	55.5
Genes with signal peptides	187	5.8
Genes with transmembrane helices	791	24.5
CRISPR repeats

**Table 4:** Number of genes associated with general COG functional categories

Code	Value	%age	Description
J	152	4.8	Translation, ribosomal structure and biogenesis
A	0	0.0	RNA processing and modification
K	132	4.2	Transcription
L	263	8.3	Replication, recombination and repair
B	0	0.0	Chromatin structure and dynamics
D	33	1.0	Cell cycle control, Cell division, chromosome partitioning
V	74	2.3	Defense mechanisms
T	139	4.4	Signal transduction mechanisms
M	123	3.9	Cell wall/membrane biogenesis
N	102	3.2	Cell motility
U	41	1.3	Intracellular trafficking and secretion
O	85	2.3	Posttranslational modification, protein turnover, chaperones
C	114	3.6	Energy production and conversion
G	223	7.0	Carbohydrate transport and metabolism
E	156	4.9	Amino acid transport and metabolism
F	57	1.8	Nucleotide transport and metabolism
H	57	1.8	Coenzyme transport and metabolism
I	41	1.3	Lipid transport and metabolism
P	119	3.7	Inorganic ion transport and metabolism
Q	15	0.5	Secondary metabolites biosynthesis, transport and catabolism
R	313	9.8	General function prediction only
S	170	5.4	Function unknown
-	1115	35.2	Not in COGs

The total is based on the total number of protein coding genes in the genome

## Insights from the genome sequence

### Potential pathogenicity related factors

Putative pathogenicity related proteins that are present in the genomes of *T. pallidum* [29] and *T. denticola* [30] were predicted in ‘*T. phagedenis**’* strain V1. Protein sequences from *T. pallidum* strain Nichols (accession number NC_000919) and *T. denticola* strain ATCC 35405 (accession number NC_002967) were used to perform blast searches against the predicted proteins of '*T. phagedenis**'* V1. These contained genes that encode for putative adhesins, antigens and a major sheath protein (Additional file 2: Table S2). Also, 22 CDS encoding chemotaxis and motility proteins, 17 CDS encoding transposases, 2 CDS encoding hemolysins and 3 putative prophages were predicted in the '*T. phagedenis**'* genome annotation.

Lipoproteins are considered to be of special attention in spirochetes because of their abundance in different spirochetal genera including *Treponema* [31]. Several of them localize to the bacterial surface and are considered as important vaccine targets. Lipoprotein prediction was thus performed separately using the SpLip server [32] that predicted 155 probable lipoproteins. The predicted lipoproteins were then Blasted against the proteins in all bacteria. Two lipoproteins with homology to known virulence related or antigenic proteins in other treponemes were expressed in *Escherichia coli* and are being used in ongoing studies.

## Conclusions

The genome sequence of ‘*T. phagedenis**’* strain V1 provides useful information on potential virulence related and antigenic proteins, which may help to establish the role of treponemes in digital dermatits in cattle. They may also be used in development of diagnostic tools and prevention strategies for the disease. Comparative studies with genome sequences of treponemes in general and ‘*T. phagedenis**’* isolates from digital dermatitis lesions in particular, can be performed. The V1 genome sequence may also prove useful for classification purposes.

## Additional files

Additional file 1: Table S1. Assembly statistics for different libraries. (DOC 27 kb) Additional file 2: Table S2. Putative pathogenicity related proteins in *T. denticola* strain ATCC 35405 and *T. pallidum* subsp. *pallidum* strain Nichols with homologues in ‘*T. phagedenis’* V1. (DOC 33 kb)
