# Supplementary material for: Draft genome sequence of ‘Treponema phagedenis’ strain V1, isolated from bovine digital dermatitis
Source: Stand Genomic Sci. 2015 Sep 21;10:67. doi: 10.1186/s40793-015-0059-0 (PMC4576374; doi:10.1186/s40793-015-0059-0)
Supplement: Additional file 1: Table S1. — Assembly statistics for different libraries. (DOC 27 kb) [file 40793_2015_59_MOESM1_ESM.doc]

| Assembly Name^1^ | Average coverage | Number of contigs | N50 contig size | Largest contig size |
| --- | --- | --- | --- | --- |
| Sample160_454 | 130× | 369 | 23,201 | 103,245 |
| Sample305_454 | 100× | 290 | 33,336 | 91,678 |
| Sample505_454 | 70× | 613 | 11,322 | 57,684 |

^1^Sample160, 305 and 505 refers to the reads from Illumina sequencing libraries with corresponding insert size and 454 refers to the reads from 454 sequencing.
