# Supplementary material for: Draft genome sequence of ‘Treponema phagedenis’ strain V1, isolated from bovine digital dermatitis
Source: Stand Genomic Sci. 2015 Sep 21;10:67. doi: 10.1186/s40793-015-0059-0 (PMC4576374; doi:10.1186/s40793-015-0059-0)
Supplement: Additional file 2: Table S2. — Putative pathogenicity related proteins in T. denticola strain ATCC 35405 and T. pallidum subsp. pallidum strain Nichols with homologues in ‘T. phagedenis’ V1. (DOC 33 kb) [file 40793_2015_59_MOESM2_ESM.doc]

| ‘*[T. phagedenis](http://dx.doi.org/10.1601/nm.26082#_blank)*’ V1 protein locus_tag | *[Treponema](http://dx.doi.org/10.1601/nm.7857#_blank)* spp protein locus_tag^1^ | Gene product | Amino acid identity (%) |
| --- | --- | --- | --- |
| TPHV1 _10302 | TP0326 | Antigen | 56 |
| TPHV1_20066 | TP0453 | Antigen | 40 |
| TPHV1_40181 | TP0751 | Laminin-binding protein | 42 |
| TPHV1_510060 | TP0155 | Fibronectin-binding protein | 58 |
| TPHV1_290003 | TP0136 | Fibronectin binding protein | 37 |
| TPHV1_40016 | TP0487 | Antigen | 59 |
| TPHV1_10302 | TP0971 | Membrane antigen, pathogen-specific Tpd) | 58 |
| TPHV1_190050 | TP0257 | Glycerophosphodiester phosphodiesterase (Gpd) | 60 |
| TPHV1_100034 | TDE_0405 | Major outer sheath protein | 38 |
| TPHV1_130036 | TDE_2258 | Surface antigen BspA | 55 |
| TPHV1_60100 | TDE_2056 | Hemin Binding Protein A (HbpA) | 49 |
| TPHV1_30021 | TDE_0842 | Cytoplasmic filament protein A (CfpA) | 82 |

^1^ locus_tag starting with TDE refers to the *[T. denticola](http://dx.doi.org/10.1601/nm.7862#_blank)* protein, locus tag starting with TP refers to *[T. pallidum](http://dx.doi.org/10.1601/nm.7858#_blank)* subsp. *pallidum* protein.
